# Supplementary material for: The development of a safe opioid use agreement for surgical care using a modified Delphi method
Source: PLoS One. 2023 Sep 26;18(9):e0291969. doi: 10.1371/journal.pone.0291969 (PMC10522037; doi:10.1371/journal.pone.0291969)
Supplement: S1 File — Participants completed the Round 1 and Round 3 surveys virtually through a secure online platform. (PDF) [file pone.0291969.s001.pdf]

Opioid Agreement Delphi Questionnaire

Your name: (This will only be visible to the research team. It will allow us to provide your responses back to you for future survey rounds. Your responses will remain anonymous to other participants)

What is your role at [hospital]?

For each statement, please indicate the comprehensibility for patients and care providers and the importance of including it in the agreement.

	Importance of including this statement	Comprehensibility of the statement	Feedback/ explanations (if any)
1. I understand that an opioid is a medication to treat pain.	1. Not important<br>2. Slightly important<br>3. Moderately important<br>4. Important<br>5. Very important	1. Very poor<br>2. Poor<br>3. fair<br>4. Good<br>5. Very good	*Free text, no limits*
<u>How I will use pain medications prescribed for this surgery</u>			
2. I will communicate fully with my doctor about the intensity of my pain, the effect of the pain on my daily life, and how well the medicine is helping to relieve the pain.	1. Not important<br>2. Slightly important<br>3. Moderately important<br>4. Important<br>5. Very important	1. Very poor<br>2. Poor<br>3. fair<br>4. Good<br>5. Very good	*Free text, no limits*
3. I agree that I will use my medicine as prescribed. If I use my medicine at a greater rate it could lead to drug overdose causing severe sedation and respiratory depression and death.	1. Not important<br>2. Slightly important<br>3. Moderately important<br>4. Important<br>5. Very important	1. Very poor<br>2. Poor<br>3. fair<br>4. Good<br>5. Very good	*Free text, no limits*
4. I will inform my doctor of all medications I am taking, including any herbal/health supplements.	1. Not important<br>2. Slightly important<br>3. Moderately important<br>4. Important<br>5. Very important	1. Very poor<br>2. Poor<br>3. fair<br>4. Good<br>5. Very good	*Free text, no limits*
5. I understand that there can be serious side effects when I use the opioid medications when I am taking other medications, such as Valium or Ativan; other opioid medicines; sedatives such as Soma, Xanax, Fiorinal; antihistamines like Benadryl; herbs, alcohol, and cough syrup containing alcohol, codeine, or hydrocodone.	1. Not important<br>2. Slightly important<br>3. Moderately important<br>4. Important<br>5. Very important	1. Very poor<br>2. Poor<br>3. fair<br>4. Good<br>5. Very good	*Free text, no limits*
<u>How I will store my pain medications prescribed for this surgery</u>			
6. I will safeguard my pain medication from loss, theft, or unintentional use by others.	1. Not important<br>2. Slightly important<br>3. Moderately important<br>4. Important	1. Very poor<br>2. Poor<br>3. fair<br>4. Good<br>5. Very good	*Free text, no limits*

	5. Very important		
7. I understand that lost or stolen medications will not be replaced.	1. Not important<br>2. Slightly important<br>3. Moderately important<br>4. Important<br>5. Very important	1. Very poor<br>2. Poor<br>3. fair<br>4. Good<br>5. Very good	*Free text, no limits*
8. I understand that the pain medication is strictly for my own use. I will never share my medication with anyone because it may endanger that person's health and is against the law	1. Not important<br>2. Slightly important<br>3. Moderately important<br>4. Important<br>5. Very important	1. Very poor<br>2. Poor<br>3. fair<br>4. Good<br>5. Very good	*Free text, no limits*
<u>How I will dispose of any unused pain medications prescribed for this surgery</u>			
9. I will dispose of unused opioid medicines as recommended by my doctor or pharmacy when I am done using them to treat my pain from surgery.	1. Not important<br>2. Slightly important<br>3. Moderately important<br>4. Important<br>5. Very important	1. Very poor<br>2. Poor<br>3. fair<br>4. Good<br>5. Very good	*Free text, no limits*
10. I understand that my doctor is required by law to check the state database, which lists other opioid prescriptions that I receive before writing a new prescription for an opioid medicine.	1. Not important<br>2. Slightly important<br>3. Moderately important<br>4. Important<br>5. Very important	1. Very poor<br>2. Poor<br>3. fair<br>4. Good<br>5. Very good	*Free text, no limits*
11. Are there any other topics or statements that are not covered in this list, that you believe should be included?	1. No<br>2. Yes, _________________ (free text)		
12. Do you have any additional comments or ideas for the use of an opioid agreement to increase safe opioid use following surgery?	*Free text, no limits*		
