# Supplementary material for: The development of a safe opioid use agreement for surgical care using a modified Delphi method
Source: PLoS One. 2023 Sep 26;18(9):e0291969. doi: 10.1371/journal.pone.0291969 (PMC10522037; doi:10.1371/journal.pone.0291969)
Supplement: S2 File — After each round, participants received an individualized feedback report that compared their responses to the summary of responses from the Delphi panel. (PDF) [file pone.0291969.s002.pdf]

Summary of feedback on Safe Opioid Use Agreement for acute pain

Survey responses from [Participant Name]

The table below shows your individual responses to the survey on the Safe Opioid Agreement for acute pain, as well as the responses from others. During the discussion session on Tuesday July 28, we will discuss all findings and potential improvements with selected panel members.

**Safe opioid use agreement Statement**	**Your responses**		**Responses from all panel members**
	**Importance**	**Comprehensibility**	
1. I understand that an opioid is a medication to treat pain.	Very important	Very good	100% stated this statement is important or very important<br>92% stated this statement's comprehensibility good or very good
2. I will communicate fully with my doctor about the intensity of my pain, the effect of the pain on my daily life, and how well the medicine is helping to relieve the pain.	Very important	Very good	86% stated this statement is important or very important<br>78% stated this statement's comprehensibility good or very good<br>*Feedback from panel:*<br>• "May be hard to conceptualize, especially in advance of using the medication."<br>• "How is the patient supposed to do this? When?"
3. I agree that I will use my medicine as prescribed. If I use my medicine at a greater rate it could lead to drug overdose causing severe sedation and respiratory depression and death	Very important	Very good	100% stated this statement is important or very important<br>68% stated this statement's comprehensibility good or very good<br>*Feedback from panel:*<br>• "Overly technical language", "wording to complex"<br>• "My only concern was the use of respiratory depression."<br>• "The first sentence is not really needed, since the first part of the second sentence is essentially a more precise version of the first. Could be

Safe opioid use agreement Statement	Your responses		Responses from all panel members
	Importance	Comprehensibility	
			shortened to make it more comprehensible. Also, the use of "medicine" and "drug" in the same paragraph might be confusing to some patients."<br>• "A better explanation of drug overdose symptoms"
4. I will inform my doctor of all medications I am taking, including any herbal/health supplements.	Very important	Very good	86% stated this statement is important or very important<br>89% stated this statement's comprehensibility good or very good<br>*Feedback from panel:*<br>• "add the term "over-the-counter pain medications and other prescription pain medications""
5. I understand that there can be serious side effects when I use the opioid medications when I am taking other medications, such as Valium or Ativan; other opioid medicines; sedatives such as Soma, Xanax, Fiorinal; antihistamines like Benadryl; herbs, alcohol, and cough syrup	Very important	Very good	83% stated this statement is important or very important<br>62% stated this statement's comprehensibility good or very good<br>*Feedback from panel:*<br>• "This sentence is too wordy. Exchange the second "when" in line 2 to "if". It would be much better to list the class/medications as bullet points."<br>• "What type of side effects?"<br>• "Mix of generic and brand names is confusing"<br>• "The examples of each medication are essential but I think the statement may be difficult to read with so many ; and

**Safe opioid use agreement Statement**	**Your responses**		**Responses from all panel members**
	**Importance**	**Comprehensibility**	
			fragments for non-health care people.”
6. I will safeguard my pain medication from loss, theft, or unintentional use by others.	Very important	Very good	94% stated this statement is important or very important<br>84% stated this statement’s comprehensibility good or very good<br>*Feedback from panel:*<br>• “Define what is meant by safeguard so it is very clear what the patient is required to do. For example, keep securely locked out of reach of others.”
7. I understand that lost or stolen medications will not be replaced.	Very important	Very good	89% stated this statement is important or very important<br>86% stated this statement’s comprehensibility good or very good<br>*Feedback from panel:*<br>• “As a pharmacist, I have seen patients not fully aware of the controlled substance laws in regards to 'refill too soon', etc”<br>• “Add the word "opioid" before the word "medications". The sentence will lead to confusion as other lost medications such as an antibiotic or stool softener, may be prescribed again.”<br>• “Not sure this is true”
8. I understand that the pain medication is strictly for my own use. I will never share my medication with anyone because it may endanger that person’s health and is against the law	Very important	Very good	94% stated this statement is important or very important<br>95% stated this statement’s comprehensibility good or very good

**Safe opioid use agreement Statement**	**Your responses**		**Responses from all panel members**
	**Importance**	**Comprehensibility**	
9. I will dispose of unused opioid medicines as recommended by my doctor or pharmacy when I am done using them to treat my pain from surgery.	Very important	Very good	89% stated this statement is important or very important<br>76% stated this statement's comprehensibility good or very good<br>*Feedback from panel:*<br>• "For patients this is a fairly new concept, safe disposal and may need direction and or reminders in regards to safe disposal."<br>• "Disposal method, place, and time, must be provided at the time of prescription."<br>• "This is good, but a bit vague. How do I determine when I am "done" with the medication? Will I need to keep it around for a while in case my pain flares up? And will I remember the instructions my doctor / pharmacist provided a few weeks prior?"<br>• "I have witnessed many patients keeping controlled substances or other medications on hand or around the house from previous years."
10. I understand that my doctor is required by law to check the state database, which lists other opioid prescriptions that I receive before writing a new prescription for an opioid medicine.	Very important	Very good	72% stated this statement is important or very important<br>76% stated this statement's comprehensibility good or very good<br>*Feedback from panel:*<br>• "Many patients don't know state database exists."

Safe opioid use agreement Statement	Your responses		Responses from all panel members
	Importance	Comprehensibility	
			• “Lack of understanding of what the database is would be my biggest concern”<br>• “Suggest simplifying to say "I understand that my doctor is required to confirm all opioid prescriptions I have received before giving new prescriptions.”<br>• “Explain possible consequences of this to the patient.”<br>• “Do doctors actually do this right now?”
