# Supplementary material for: The development of a safe opioid use agreement for surgical care using a modified Delphi method
Source: PLoS One. 2023 Sep 26;18(9):e0291969. doi: 10.1371/journal.pone.0291969 (PMC10522037; doi:10.1371/journal.pone.0291969)
Supplement: S3 File — A document was drafted to address the additional patient education needs identified by the participants. (PDF) [file pone.0291969.s003.pdf]

Educational markers for surgery opioid agreement for clinicians

Statement	Ensure that education is provided on
1. I understand that an opioid is a medicine used to treat pain.	○ What an opioid is<br>○ *Optional: write in medicine name here (depends on when opioid agreement is administered)*
<u>Patient's medication use</u>	
2. I will tell my doctor about my pain.	○ *When* and *how* to communicate with *whom* on pain
3. I understand that if I use more of my opioid pain medicine than prescribed it could cause an overdose and death.	○ Why overdose is dangerous and what are symptoms<br>○ When to call 911 or go to ED
4. I will tell my doctor all of the medicines I am taking, including any herbal/health supplements.	-
5. I understand that there can be serious side effects if I use my opioid pain medicine while I am using other medicines or substances, such as:<br>□ Other opioid pain medicines (e.g. oxycodone, codeine)<br>□ Benzodiazepine sedatives (e.g. diazepam (Valium®), lorazepam (Ativan®), alprazolam (Xanax®))<br>□ Muscle relaxants (e.g. carisoprodol (Soma®), cyclobenzaprine (Flexeril®))<br>□ Headache pain medicine containing a butalbital (e.g. Fiorinal®)<br>□ Antihistamines (e.g. diphenhydramine (Benadryl®))<br>□ Cough suppressants<br>□ Alcohol<br>□ Herbal supplements	○ This also applies to new medications prescribed after discharge<br>○ Individualized examples may be helpful (e.g. a beer or glass of wine, sleeping pills)<br>○ Who to contact if questions about potential interactions<br>○ Tapering if relevant
<u>Medication storage</u>	
6. I will keep my opioid pain medicine safely stored to avoid loss, theft or use by others.	○ What would safe storage look like? (e.g. locked)
7. I understand that lost or stolen opioid pain medicine may not be replaced.	-
9. I understand that the opioid pain medicine is strictly for my own use. I will **never** share my opioid medicine with anyone because it may harm that person's health, and it is **against the law.**	-
<u>Medication disposal</u>	
9. I will safely dispose of the unused opioid medicine when I am done using it to treat my pain from this surgery.	○ When can the patient consider to be done<br>○ How and where can to safely dispose
<u>IL-PMP</u>	
10. I understand that my doctor is required by law to check the state records for other opioid prescriptions that I receive before writing a new opioid pain medicine prescription for me.	○ Explain that this may mean that they may not get a new prescription if they already have one
