# Supplementary material for: The development of a safe opioid use agreement for surgical care using a modified Delphi method
Source: PLoS One. 2023 Sep 26;18(9):e0291969. doi: 10.1371/journal.pone.0291969 (PMC10522037; doi:10.1371/journal.pone.0291969)
Supplement: Attachment — Submitted filename: PONE_D-23-06761_R1_2.docx [file pone.0291969.s005.docx]

The manuscript has improved significantly and most comments from the previous peer-review have been carefully handled. However, there are still some issues in the method section that need to be improved.

**Method**

Page 5-7, Rounds of Delphi study, I recommend that you use headlines in this section as it is a bit hard to follow the methods in the current format. Suggestions of headlines are for example ***Preparations*** (where you describe how the first version of the agreement were developed), ***Pilot testing***, ***Delphi Round 1***, ***Delphi round 2***, and ***Delphi round 3***.

Page 5, line 106-111. The addition concerning that the authors developed the draft agreement is good but to understand the process it would be good with more info concerning why these agreements were used, why you choose the specific items and how you reached agreement in the group.

Page 5, section 2. Here you write about a pilot study. As this isn’t part of the Delphi round this section is a bit unclear. Why did you do this pilot testing before the Delphi rounds? Is it suggested in the literature?

Page 7, data analysis. This section is a bit confusing, you write that you used descriptive statistics and then summarized the qualitative responses from the free text fields. So were the thematic analysis made just from Delphi round 1 and 3 where the participant answered the survey in free text? If not this must be clarified. If yes, how did you analyze round 2, in the qualitative interviews?  What did you do with the audio recordings from the interviews? Were they transcribed and analyzed? This must be clarified both for the reader and for the replicability.

Page 7. How was the thematic analyses made, add info about the steps.

Table 3. I like the addition of Table 3 as it contributes with info about the results of the thematic analysis. It would be good with more info about the analysis step and it could be helpful with an example of how the analysis was made from full text to a theme. For example there seems to be some overlap between the themes, for example formatting and connotation of certain words could perhaps be part of comprehensibility? How did you argue when you did the analyses and how do you handle the overlaps.
