# Supplementary material for: The development of a safe opioid use agreement for surgical care using a modified Delphi method
Source: PLoS One. 2023 Sep 26;18(9):e0291969. doi: 10.1371/journal.pone.0291969 (PMC10522037; doi:10.1371/journal.pone.0291969)
Supplement: Attachment — Submitted filename: OpioidAgreementDelphi_ResponsetoReviewers_V2.docx [file pone.0291969.s006.docx]

**Responses to Reviewer Comments (PONE-D-23-06761R1)**

**Reviewer #2**

General Comment: The manuscript has improved significantly and most comments from the previous peer-review have been carefully handled. However, there are still some issues in the method section that need to be improved.

**RESPONSE:** Thank you very much for your time and thorough review of our revised manuscript. We appreciate the additional feedback and have addressed each point below.

1. Page 5-7, Rounds of Delphi study, I recommend that you use headlines in this section as it is a bit hard to follow the methods in the current format. Suggestions of headlines are for example ***Preparations*** (where you describe how the first version of the agreement were developed), ***Pilot testing, Delphi Round 1, Delphi round 2, and Delphi round 3.***

**RESPONSE:** Thank you for this suggestion. We have added sub-headings in the methods section to make it easier to follow.

**Revision to manuscript: “*Pilot testing***” **(pg. 5, line 120); “*Round 1***” **(pg. 6, line 129); “*Round 2***” **(pg. 6, line 135); “*Round 3***” **(pg. 7, line 163)**

2. Page 5, line 106-111. The addition concerning that the authors developed the draft agreement is good but to understand the process it would be good with more info concerning why these agreements were used, why you choose the specific items and how you reached agreement in the group.

**RESPONSE:** We have added some more information about the process of developing the draft agreement for the Round 1 survey. The sample opioid agreements cited in the text were chosen based on what was publicly available and their applicability to the surgical setting. Prior surgical opioid reduction initiatives within our healthcare system guided our research team in understanding what was needed in an opioid use agreement for surgery and choosing items (e.g., including disposal of leftover opioids). Our research team discussed the publicly available agreements, items important for the surgical setting, and developed the content for draft agreement through group discussions. This then served as a starting point for the Delphi study where participants were asked if the items were important to include and if there were other topics, not in the original draft agreement, that should be included as well.

**Revision to manuscript: “**Items included in the draft agreement were chosen through discussions within the research team and were based on publicly available opioid agreements and our previous research on surgical opioid reduction within our health system. For example, our previous work showed that communicating and setting expectations about pain relief as well as discussing safe disposal of leftover opioids were important areas for improvement, so they were included in the draft agreement (48, 49).” **(pg. 5, lines 108-112).**

3. Page 5, section 2. Here you write about a pilot study. As this isn’t part of the Delphi round this section is a bit unclear. Why did you do this pilot testing before the Delphi rounds? Is it suggested in the literature?

**RESPONSE:** Thank you for this comment. As we had previously mentioned in the discussion section (pg. 19, line 342), pilot testing the survey is a recommended part of a Delphi study. We have clarified this in the methods section and added the citations to support conducting a pilot study before the first round of the Delphi study.

**Revision to manuscript: “**Pilot testing is recommended before starting a Delphi study (41, 48).” **(pg. 5, lines 121).**

4. Page 7, data analysis. This section is a bit confusing, you write that you used descriptive statistics and then summarized the qualitative responses from the free text fields. So were the thematic analysis made just from Delphi round 1 and 3 where the participant answered the survey in free text? If not this must be clarified. If yes, how did you analyze round 2, in the qualitative interviews? What did you do with the audio recordings from the interviews? Were they transcribed and analyzed? This must be clarified both for the reader and for the replicability.

**RESPONSE:** We have clarified that we recorded and transcribed the Round 2 focus group. We conducted the same process of thematic analysis for the qualitative data from all three rounds (free text for Rounds 1 and 3 and transcribed from the focus group in round 2) and have clarified this in the text.

**Revision to manuscript: “**The focus group lasted 90 minutes, was conducted virtually, and was audio recorded and transcribed for analysis.” **(pg. 6, lines 138-139).**

**Revision to manuscript: “**Survey responses were exported from Qualtrics (Qualtrics, Provo, UT) to Microsoft Excel (Microsoft Corporation, Version 2304) and quantitative values were summarized using descriptive statistics. Qualitative responses from the free text fields in the Round 1 and Round 3 surveys as well as the transcript of the focus group from Round 2 were summarized using a simple thematic analysis approach and lean coding (52, 53). First, two researchers (CBI & WLS) reviewed all qualitative comments provided on the Round 1 and Round 3 surveys and the transcript from the focus group. Second, the two researchers discussed the qualitative data to develop codes inductively which were applied to the data through group discussion. For example, quotes relating to desired reading level of the items were labeled as “reading level”. Third, they created a table grouping the broad codes to identify overarching themes (52). For example, the “reading level” code was assigned to the theme “comprehensibility”. Finally, themes and example quotes were discussed with two additional team members and refined for clarity (JKJ & SNB).” **(pg. 7-8, lines 172-183).**

5. Page 7. How was the thematic analyses made, add info about the steps.

**RESPONSE:** Please see comment 6 below.

6. Table 3. I like the addition of Table 3 as it contributes with info about the results of the thematic analysis. It would be good with more info about the analysis step and it could be helpful with an example of how the analysis was made from full text to a theme. For example there seems to be some overlap between the themes, for example formatting and connotation of certain words could perhaps be part of comprehensibility? How did you argue when you did the analyses and how do you handle the overlaps.

**RESPONSE:** We have provided a more detailed description of the four-step process we conducted, including reviewing the data, developing codes, categorizing codes into themes, and receiving feedback on the final themes.

**Revision to manuscript: “**Qualitative responses from the free text fields in the Round 1 and Round 3 surveys as well as the transcript of the focus group from Round 2 were summarized using a simple thematic analysis approach and lean coding (52, 53). First, two researchers (CBI & WLS) reviewed all qualitative comments provided on the Round 1 and Round 3 surveys and the transcript from the focus group. Second, the two researchers discussed the qualitative data to develop codes inductively which were applied to the data through group discussion. For example, quotes relating to desired reading level of the items were labeled as “reading level”. Third, they created a table grouping the broad codes to identify overarching themes (52). For example, the “reading level” code was assigned to the theme “comprehensibility”. Finally, themes and example quotes were discussed with two additional team members and refined for clarity (JKJ & SNB).” **(pg. 7-8, lines 174-183).**
